# Backfat Thickness Does Affect the Restoration of Ovarian Activity Postpartum in Different Breeding Programs in Zebu Cattle

**DOI:** 10.3389/fvets.2021.794632

**Published:** 2021-12-09

**Authors:** José F. Martínez, Carlos S. Galina, Pablo Ortiz, Manuel D. Corro, Ivette Rubio, Juan J. Romero-Zuñiga

**Affiliations:** ^1^Faculty of Veterinary Medicine and Zootechnics, Department of Reproduction, National Autonomous University of Mexico, Mexico City, Mexico; ^2^Centre for Teaching, Research and Extension in Tropical Animal Husbandry, Faculty of Veterinary Medicine and Zootechnics, National Autonomous University of Mexico, Tlapacoyan, Mexico; ^3^Research Program in Population Medicine, Veterinary Medicine School, National University, Heredia, Costa Rica

**Keywords:** breeding program, postpartum, Brahman, tropics, fertility

## Abstract

The backfat thickness (BFT) was used to forecast the onset of ovarian activity and predict the calf growth. Eighty Brahman cows with their calves were allocated in two groups of 40 distributed in 4 months according to the month of calving, starting in March and finishing in June. One was synchronized and inseminated at fixed time following by natural mating (TAI+NM); whilst the other only by natural mating (NM). The programs started at 60 ± 5 days postpartum and ended 60 days later. From day 30 postpartum, serial ultrasound examinations and progesterone samples were used to monitor the onset of ovarian activity. The BFT in the rump area was measured by ultrasound from 30 days postpartum and every 15 days thereafter. The weight of the calves was recorded at birth and at weaning on 160 days. The adjusted effect of BFT on ovarian activity and the calves' development was assessed by binomial logistic regression at 30, 60, 75, and 120 days postpartum. The cycling cows averaged higher BFT irrespective of breeding program (*P* < 0.001). Also, slower changes in BFT were recorded during the follow-up at each time for all cows. However, the former had the higher BFT values from calving to the end of the study (*P* < 0.001). At 60, 75, and 120 days, the BFT measured, at the preceding time, was the only factor predicting the commencement of cyclicity (*P* < 0.001). The accumulative pregnancy through time was higher in TAI+NM (*P* = 0.003). Daily weight gain and weaning weight of the calves born in March was significantly heavier (*P* < 0.001) than peers born in April, May, or June. The most critical element to forecast the onset of ovarian activity is the monitoring of BFT around calving regardless of the breeding program. BFT to estimate the development of the calves until weaning was unpredictable.

## Introduction

The restoration of ovarian activity in Zebu cattle is commonly affected by the availability and quality of native or introduced pastures. These features are dependent on the onset of the rainy season, that can be unpredictable in many areas of the tropical world (1). In many instances, the poor management in the last trimester of gestation incises in the body condition of the dams, which would further accentuate the loss of body weight if the rainy season is delayed (2). Thus, combining body weight loss in the last trimester of gestation and a delayed rainy season, makes a problematic scenario for clinicians and farmers alike to achieve a successful pregnancy rate. Consequently, past and present studies have shown that about only 50% of cattle calve every year (3).

Sa-Filho et al. (4) reported that the use of synchronizing agents with timed artificial insemination and natural mating, increases the number of pregnant cows in the short term, compared only to natural mating. However, at the end of a 90-day breeding season the proportion of pregnant animals was similar. The authors also observed that the number of pregnant animals was highly associated with body condition. Ultrasonographic measurements of backfat thickness (BFT) is a tool allowing us to obtain an objective measure of the animal current metabolic state, especially in the last trimester of gestation, when other indicators such as weight, body condition score, glucose, non-esterified fatty acids (NEFA's), among others, become difficult to interpret (2, 5, 6). This methodology is not frequently used in breeding programs for predicting the early resumption of ovarian activity postpartum. Furthermore, the development of the calves in the tropics can add useful information in the decision-making process as to decide on the most adequate time to breed animals (7).

The objective of the present study was to compare, the effect of backfat thickness after calving on the resumption of ovarian activity, and calf birth weight to weaning, testing two different breeding programs.

## Materials and Methods

### Location

The study was conducted, from March to June, at the Center for Teaching, Research and Extension in Tropical Animal Husbandry belonging to the Faculty of Veterinary Medicine and Zootechnics of the National Autonomous University of Mexico, located in the State of Veracruz, Mexico at 20° 04′N and 97° 03′W, with a humid tropical climate, mean annual temperature of 24°C and mean annual rainfall of 1,742 mm.

### Animals

A total of 80 multiparous postpartum Brahman cows between 4 and 10 years in a proportion of 10 animals per year of the study and their calves were used and distributed equally according to the month of calving from March to June. All animals were kept under pasture conditions based on rotational grazing of *Cynodon nlemfuensis* (African star grass), *Paspalum spp*. and *Axonopus pp*, supplemented with minerals and water *ad libitum*.

### Experimental Design

**Figure 1:**
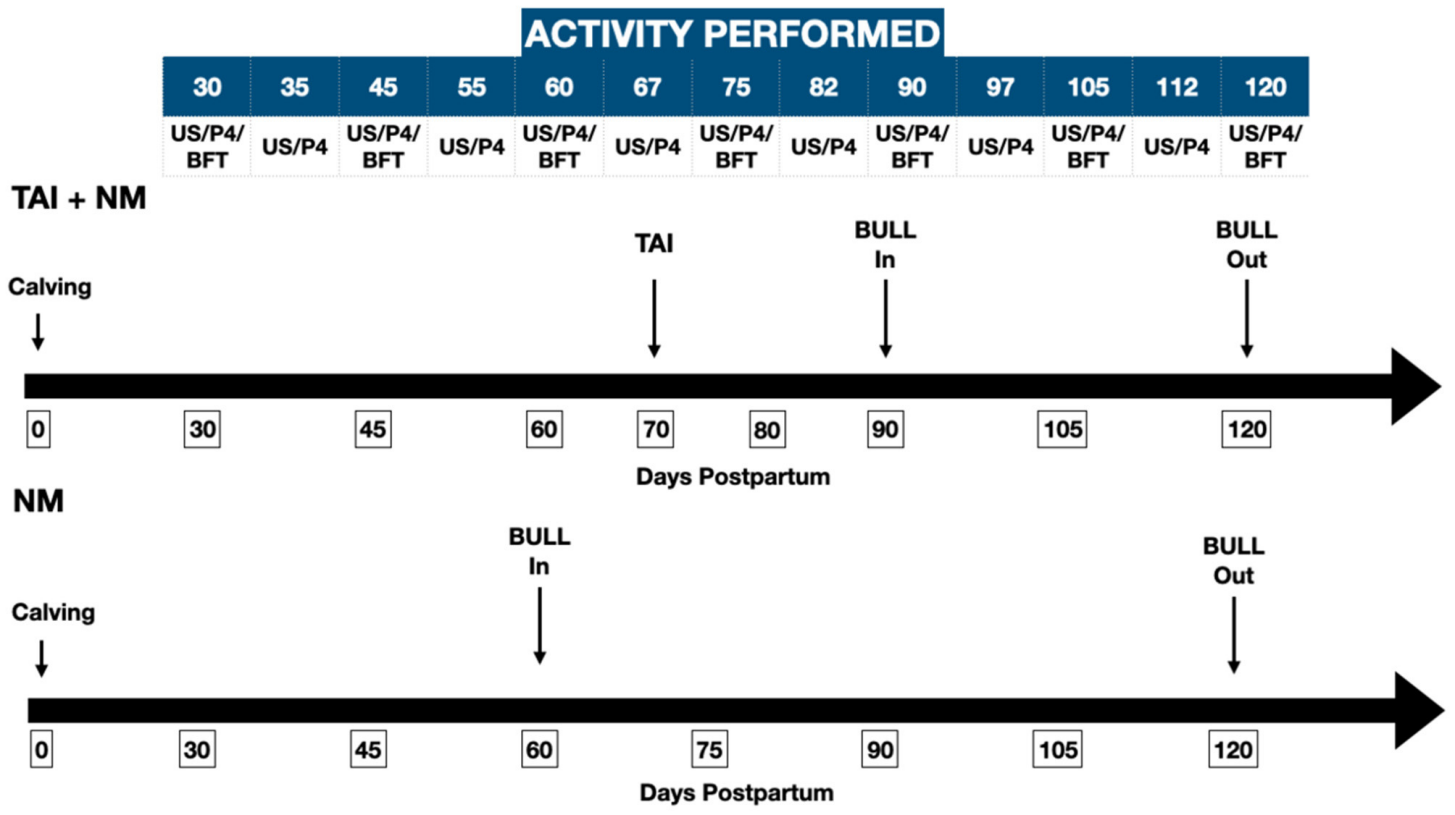
Chronology of the sequence of events and activities performed in TAI+NM (timed artificial insemination followed by natural mating) and NM (natural mating only) breeding programs. TAI, timed artificial insemination; NM, natural mating; US, transrectal ultrasonography; P4, progesterone samples; BFT, backfat thickness.

The 80 Brahman cows with their calves were allocated in two groups of 40 distributed in 4 months according to the month of calving, starting in March and finishing in June; one was synchronized and inseminated at a fixed time. Following this event, continuing with a bull of proven fertility introduced to the herd (TAI + NM). The other was allocated to a natural mating (NM) program using a bull with an approved breeding soundness evaluation. The programs started 60 ± 5 and ended 60 days later (Figure 1).

### Breeding Programs

The TAI + NM cows were synchronized using a protocol based on the use of a device with 1.9 g of natural progesterone (CIDR 1900 Cattle Insert, Zoetis, Mexico) and the administration of 2 mg IM of estradiol benzoate (Estradiol Benzoate, Zoetis, Mexico) on the day of insertion (60-day postpartum). At CIDR withdrawal (67-day postpartum), 400 UI IM of ECG were given (Novormon 5000, Zoetis, Mexico) together with 25 mg IM dinoprost tromethamine (Lutalyse, Zoetis, Mexico). This treatment was followed by 1 mg estradiol benzoate at day 68 postpartum. Thus, artificial insemination with the same commercial Brahman bull was performed at 56 ± 2 h after CIDR withdrawal. Then, 20 days after timed artificial insemination the cows were relocated to a natural mating program with a different bull.

The NM cows remained with a 5-year Brahman bull with proven fertility and acceptable following the breeding soundness evaluation in a paddock from day 60 ± 5 to 120 postpartum.

### Backfat Thickness

The assessment of BFT in the rump area was carried out using an ultrasound device (Aloka SSD 500, Tokyo, Japan) with a convex transducer and 3.5 MHz frequency to obtain the ultrasonographic images used to measure BFT in centimeters. Observations initiated at day 30 postpartum and thereafter every 15 days. After animal immobilization, the examination site to measure BFT was the rump area, located midway between the tuber coxae (hooks) and the tuber ischiae (pins), 2–3 cm above the greater trochanter of the femur (8).

### Transrectal Ultrasonography and Progesterone Samples

The presence of a corpus luteum over time was the indicator of restarting ovulation and was evaluated by transrectal ultrasonography using a 7.5 MHz transductor (Aloka SSD 500, Tokyo, Japan). In addition, blood samples were obtained from the coccygeal vein or artery to measure serum progesterone concentration using an ELISA kit (DRG® Progesterone ELISA, Germany). The presence of a viable corpus luteum was determined when values were above 1ng mL (9). Examinations started on day 30 postpartum and thereafter every 5–10 days until the end of the study to define, cows cycling and/or non-cycling throughout the study.

### Pregnancy Diagnosis

Pregnancy diagnosis started at 35 days after the onset of the experiment and continued every 7 days to record as closely as possible the actual time of gestation. It was carried out by transrectal ultrasonography, using an ultrasound (Aloka SSD 500, Tokyo, Japan) with a linear 7.5 MHz transductor to confirm pregnancy by the presence of an amniotic vesicle, the embryo itself, and its heartbeat.

### Calves Growth

The sex and the weight of all calves were recorded at birth. All calves remained with their dams until they were weaned at 160 days of age and weighed at this time using a S3 Weigh Scale Indicator (Datamars True-Test, TX USA).

### Statistical Analysis

Backfat thickness (BFT) and age of the females were compared using the Mann-Whitney test to determine the comparability of both breeding programs (TAI+NM and NM) at the beginning of the study. Descriptive statistics (mean, SD, median, Q1, and Q3) were calculated for the BFT at 30, 45, 60, 75, 90, 105, and 120 days postpartum globally and by breeding program. Besides, Mann-Whitney tests were performed to make comparisons between programs at each time. The proportion of cows cycling at different postpartum periods, were calculated globally and into the breeding programs at 30, 60, 75, and 120 days postpartum. The difference between them was calculated by the Fisher exact test. The Mann-Whitney test was applied to compare the BFT between cycling and not-cycling cows at each period postpartum (30, 60, 75, and 120 days) This test was also performed comparing the fluctuations in BFT at 45, 60, 75, 90, and 120 days postpartum.

To estimate the effect of the backfat thickness, age of the dam, sex, and weight of the calf at birth (BBW) and weaning (WBW), the calf daily weight gain (DWG), and the month of calving month on the restarting of ovarian activity, binomial logistic regression models were performed for the 30, 60, and 75 days postpartum. This analysis was done for each timepoint in two phases: univariate and multivariate. Variables with a *P*-value <0.25 in the univariate analysis were included in the multivariate analysis. In addition, possible confusion and interactions were tested. The Akaike information criterion (AIC) was used to select the best fit model and change the log-likelihood estimates between the new and the previous model. For this purpose, the cow's age, the birth and weaning body weight, the daily weight gain, and the backfat thickness at the points chosen for analysis, were converted from their original continuous form to a categorized one using the tertiles option. A non-parametric survival analysis by Kaplan-Meier curves was undertaken to compare cows becoming pregnant early between the breeding programs and a log-rank test was used to determine their significance.

Kruskal-Wallis test was performed to compare BBW, WBW and DGW of all calves, irrespective of the breeding program, with the month of birth. Furthermore, a non-parametric Spearman correlation was undertaken to test the association between the BFT of the dam at 30 and 120 days postpartum on the BBW, WBW and DGW of all calves.

All the statistical analyses were performed with SAS 9.4 and Jamovi ver. 1.6.23.0. A *P* < 0.05 was used as the threshold for statistical significance.

## Results

**Figure 2:**
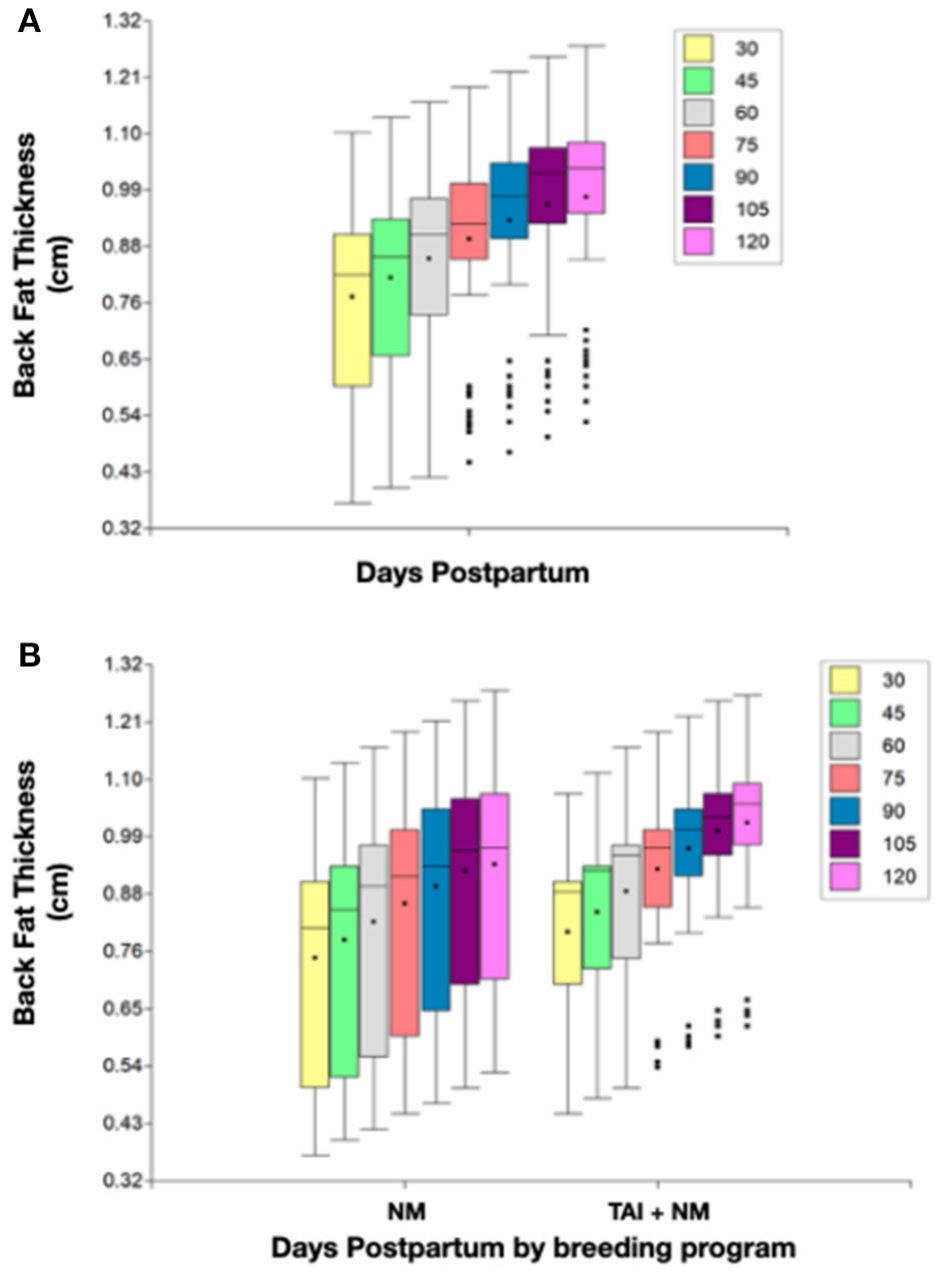
Descriptive statistics for BFT at different days postpartum for the total of cows **(A)** and by breeding program **(B)**.

No differences by age (*P* = 0.26) or BFT (H = 1.14; *P* = 0.28) were observed between breeding programs at the beginning of the follow-up. However, an ascendant tendency to gain BFT was observed in most cows, despite of the assigned program. Besides, no differences were found between breeding programs at any time (Figure 2). As can be observed, there is considerably more variation in the data of cows in the TAI+NM.

**Figure 3:**
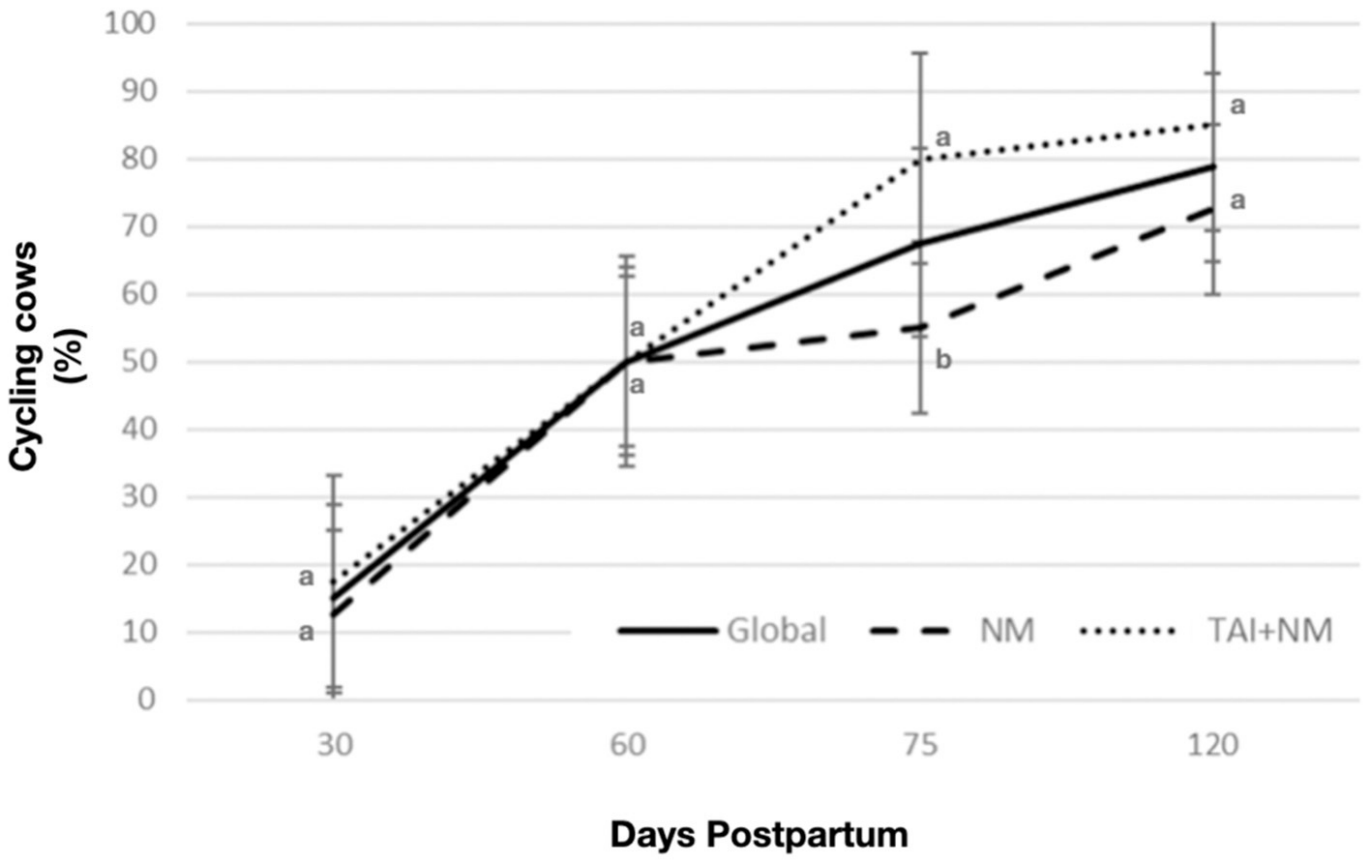
Percentages of cycling cows at different postpartum days global and by breeding program. The bar error depicts the difference between percentages. Different letters between days postpartum are statistically significant (*P* < 0.01).

A trend to increase the percentage of cycling cows is presented during the follow-up period in the breeding programs with no differences, except for the 75-day postpartum, being slightly higher (*P* < 0.05) in TAI+NM (Figure 3). On the other hand, cycling cows had a higher average of BFT in all the points of analysis irrespective of breeding program (*P* < 0.0001) (Table 1).

**Table 1:** Comparison of backfat thickness between cycling and non-cycling cows at different days postpartum.

**Day postpartum**	**Status**	***n***	**Mean**	**SD**	**W**	***P* (2 tails)**
30	Cycling	12	1.03	0.05	890.5	<0.0001
	Non-cycling	68	0.73	0.18		
60	Cycling	40	0.99	0.08	2,362.0	<0.0001
	Non-cycling	40	0.71	0.16		
75	Cycling	54	0.98	0.10	507.0	<0.0001
	Non-cycling	26	0.71	0.18		
120	Cycling	63	1.04	0.09	284.5	<0.0001
	Non-cycling	17	0.71	0.18		

The cycling cows showed significantly lower changes in BFT during the follow-up compared to the non-cycling ones (*P* < 0.001) in global and by each period, but the cycling cows had the higher values of BFT since the calving to the end of the study.

The percentage of animals cycling by 30 days postpartum, age of the cow (*P* = 0.31), month of calving (*P* = 0.66), calf sex (*P* = 1.00), BBW (*P* = 0.29), WBW (*P* = 0.31), DWG (*P* = 0.36) and breeding program (*P* = 0.53) were not associated with the restarting of cyclicity. Only the BFT in its continuous form was associated (*P* < 0.001); however, the estimated OR and its 95% CI were unpractical to interpret because of their extremely range.

By 60-day postpartum, cow's age (*P* = 0.44), month of calving (*P* = 1.00), calf sex (*P* = 0.66), the BBW (*P* = 0.63), the WBW (*P* = 0.36), the DWG (*P* = 0.45) and breeding program (*P* = 1.00) were not associated with the restarting of cyclicity. On the contrary, in the univariate analysis, the BFT measured both at the 30, 45, and 60 days postpartum, showed an increased tendency to delay the restarting of cyclicity as the BFT was lower (*P* < 0.001); however, when they are included in the multivariate model, the BFT at 45 and 60 days postpartum lost significance due to collinearity. Then, at this point, the only variable with a significant effect on the cyclicity restarting was the BFT at 30 days postpartum (Table 2).

**Table 2:** Association of the backfat thickness (BFT) and the cyclicity restarting at 60-day postpartum; univariate and multivariate results are presented.

**Univariate models**								
**Variable**	**Level**	**Estimate**	**SE**	**Z**	***P***	**Odds ratio**	**Lower**	**Upper**
BFT 30-day postpartum	Intercept	−3.22	1.02	−3.16	0.002	0.04	0.01	0.30
	0.70–0.89	2.88	1.1	2.62	0.009	17.86	2.07	154.41
	<0.70	6.59	1.44	4.57	<0.001	725.00	43.09	12199.12
*Reference level: >0.89*								
BFT 45-day postpartum	Intercept	−2.48	0.736	−3.38	<0.001	0.08	0.02	0.35
	0.75–0.92	2.17	0.836	2.6	0.009	8.80	1.71	45.32
	<0.75	5.78	1.256	4.6	<0.001	324.00	27.61	3802.33
*Reference level: >0.92*								
BFT 60-day postpartum	Intercept	−3.09	1.02	−3.02	0.003	0.05	0.01	0.34
	0.80–0.95	2.66	1.09	2.43	0.015	14.24	1.67	121.32
	<0.80	6.42	1.44	4.45	<0.001	616.00	36.44	10412.28
*Reference level: >0.95*								
**Multivariate model**								
**Variable**	**Level**	**Estimate**	**SE**	**Z**	***P***	**Odds ratio**	**Lower**	**Upper**
BFT 30-day postpartum	Intercept	−3.22	1.02	−3.16	0.002	0.04	0.01	0.30
	0.70–0.89	2.88	1.1	2.62	0.009	17.86	2.07	154.41
	<0.70	6.59	1.44	4.57	<0.001	725.00	43.09	12199.12

*Only significant associated variables are showed*.

*Model fit measures: Deviance: 66.1; AIC: 74.1; X^2^: 38.4; P < 0.001. Reference levels are the same as specified for the univariate models*.

The breeding programs become significant (*P* = 0.019) by 75-day postpartum, having the NM the higher risk of no returning to cyclicity (Univariate OR = 3.27, 95% CI: 1.21–8.84; Multivariate OR = 6.34, 95%CI: 1.74–23.12). The model for BFT at 30-day postpartum did not converge, but it does for 45, 60, and 75 days postpartum, showing a significant association between the BFT and the no return to cyclicity, having the higher risk, those cows with lower BFT (Table 3).

**Table 3:** Association of the backfat thickness (BFT) and the cyclicity restarting at 75-day postpartum; univariate and multivariate results are presented.

**Univariate analysis**								
**Variable**	**Level**	**Estimate**	**SE**	**Z**	***P***	**Odds ratio**	**Lower**	**Upper**
Breeding program	Intercept	−1.39	0.40	−3.51	<0.001	0.25	0.12	0.54
	NM	1.19	0.51	2.34	0.019	3.27	1.21	8.84
*Reference level: TAI+NM*								
BFT 45-day postpartum	Intercept	−3.22	1.02	−3.16	0.002	0.04	0.01	0.30
	0.75–0.92	2.22	1.11	2.00	0.046	9.21	1.04	81.31
	<0.75	3.81	1.09	3.48	<0.001	45.00	5.28	383.40
*Reference level: >0.92*								
BFT 60-day postpartum	Intercept	−3.09	1.02	−3.02	0.002	0.05	0.01	0.34
	0.80–0.95	1.99	1.11	1.79	0.073	7.33	0.83	64.79
	<0.80	3.58	1.09	3.28	0.001	36.00	4.24	305.83
*Reference level: >0.95*								
BFT 75-day postpartum	Intercept	−3.22	1.02	−3.16	0.002	0.04	0.01	0.30
	0.86–0.97	2.35	1.10	2.13	0.033	10.53	1.21	91.47
	<0.86	3.75	1.09	3.43	<0.001	42.50	4.97	363.16
*Reference level: >0.97*								
**Multivariate model**								
**Variable**	**Level**	**Estimate**	**SE**	**Z**	***P***	**Odds ratio**	**Lower**	**Upper**
	Intercept	−4.56	1.18	−3.87	<0.001	0.01	0.00	0.11
Breeding program	NM	1.85	0.66	2.80	0.005	6.34	1.74	23.12
BFT 45-day postpartum	0.75–0.92	2.51	1.14	2.20	0.028	12.27	1.31	114.99
	<0.75	4.35	1.17	3.73	<0.001	77.28	7.85	760.30

*Only significant associated variables are showed*.

*Model fit measures: Deviance: 66.1; AIC: 74.1; X^2^: 38.4; P < 0.001. Reference levels are the same as specified for the univariate models*.

For 120-day postpartum, the breeding programs becomes non-significant as it was for 30 and 60 days postpartum (*P* = 0.17). The model for BFT at 30-day postpartum did not converge, but it does for 45, 60, 75, 90, and 120 days postpartum, showing a significant association between the BFT and the no return to cyclicity, also, as before, having the higher risk those cows with lower BFT. When they are included in the multivariate model, the BFT at all measurements lost significance due to collinearity. None of the other adjusted variables remained in the model; thus, the best fit model was a univariate model corresponding to the BFT at 90-day postpartum.

**Figure 4:**
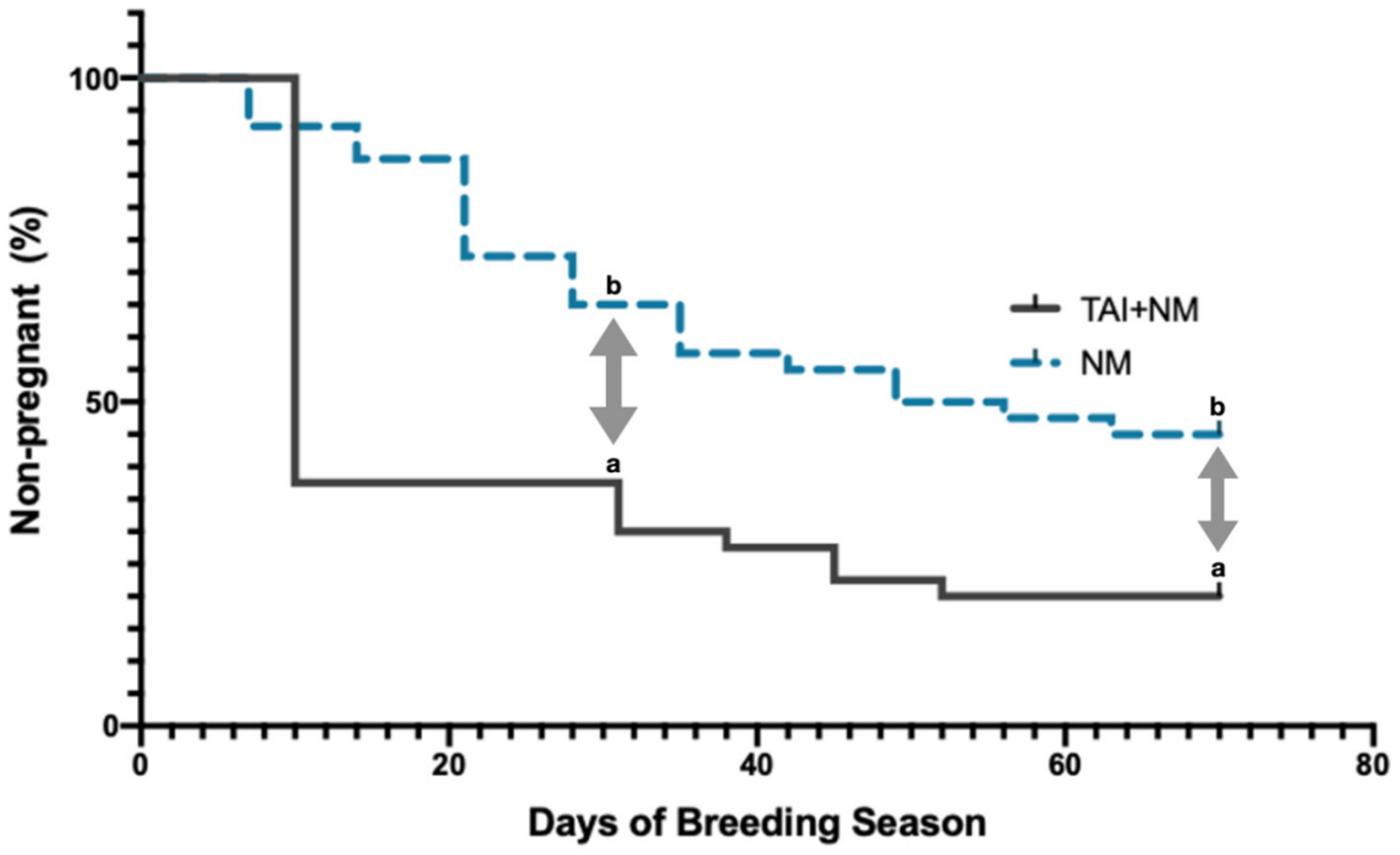
Kaplan-Meier survival curves for pregnancies by breeding program. Different letters indicated by double-headed arrows are statistically significant (*P* < 0.01).

The percentage of animals pregnant after 30 days of the breeding season was in the group for TAI+NM (25/40 = 62%) than for NM (14/40 = 35%). An overall significant difference (*P* = 0.02) was observed in the number of animals pregnant during the first 30 days. At the end of the breeding season, the percentage of animals pregnant for TAI+NM was (32/40 = 80%) and for the group of NM was (22/40 = 55%), (*P* = 0.03). The survival curves comparing the accumulative percentage of pregnancy were different between the two breeding programs (*P* = 0.003) (Figure 4).

**Figure 5:**
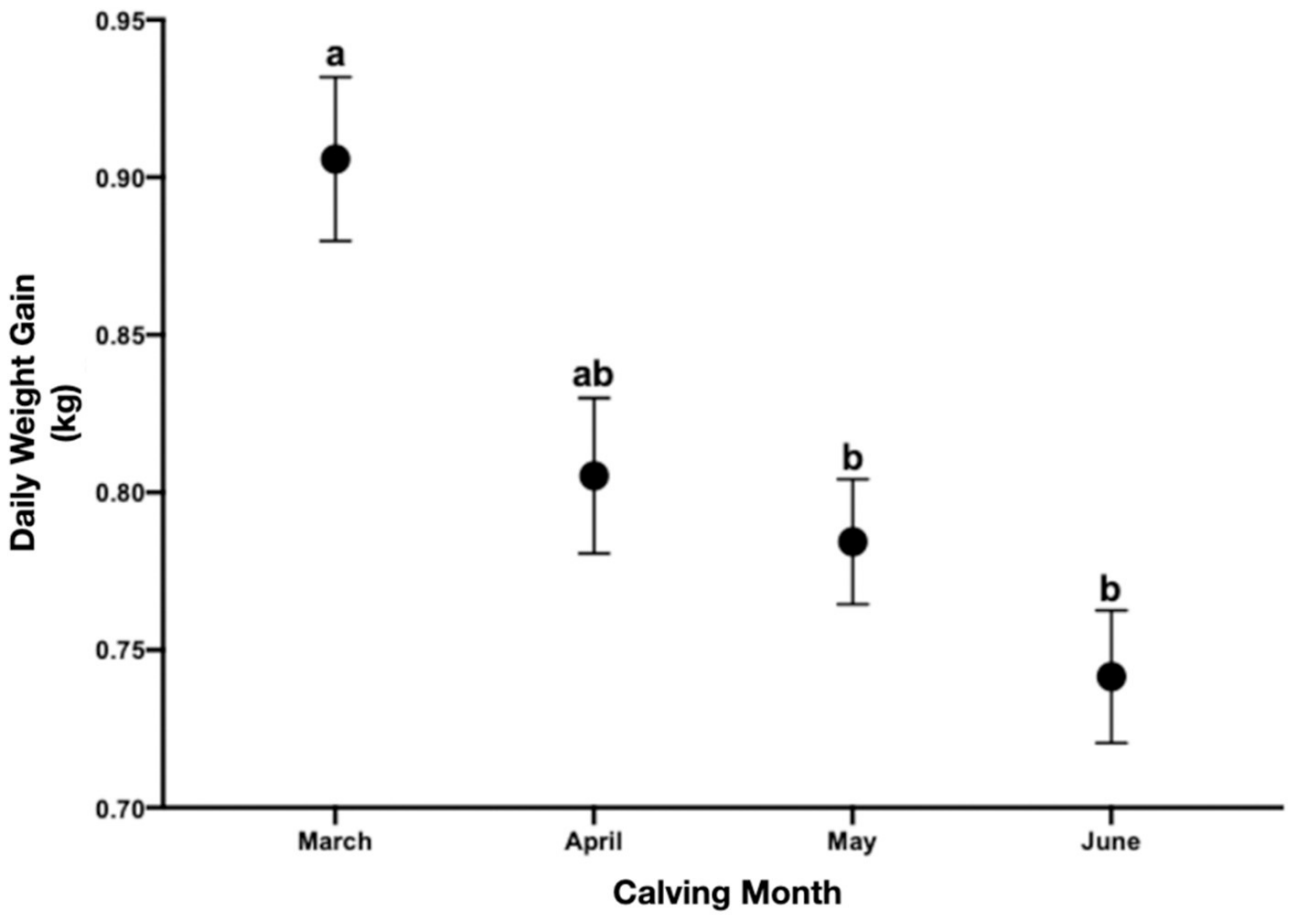
Daily weight gain of the calves at 160 days postpartum for every calving month. Different letters between months are statistically significant (*P* < 0.01).

The birth body weights by month of calving (March = 31.65 ± 3.167; April = 32.25 ± 2.673; May = 30.00 ± 5.047; June = 31.80 ± 3.847) were not significantly different (*P* > 0.05). Weaning weights by calving month (March = 176.6 ± 19.48; April = 161.1 ± 18.22; May = 155.5 ± 15.16; June = 150.5 ± 14.26) were significant (*P* < 0.0001) between March and the rest. Daily weight gains by calving month (March = 0.9058 ± 0.1162; April = 0.8053 ± 0.1101; May = 0.7844 ± 0.08860; June = 0.7416 ± 0.09405) were statistically different (*P* = 0.01 and *P* = 0.0003) for the month of March vs. May and June, respectively. April was not different to March but equal to the other months (Figure 5). At 30-day postpartum there was no correlation between BFT and BBW (r: −0.014, *P* = 0.904), WBW (r: 0.039, *P* = 0.7*3*0) and DWG (*r*: 0.027, *P* = 0.813). Neither, at the last point of analysis (day 120 postpartum) there was no correlation between BFT and BBW (*r*: 0.157, *P* = 0.165), WBW (r: 0.020, *P* = 0.860) and DWG (r: 0.006, *P* = 0.954).

## Discussion

As expected, non-significant differences were observed when analyzing the two breeding programs concerning BFT. This result matches similar studies measuring body condition scores (10) or BFT (6). By day 30 postpartum, the percentage of cows starting ovarian activity was 15% in both breeding programs, again, in agreement with several studies demonstrating the tardiness on the onset of ovarian activity, mainly if the offspring is present (11). By day 60 postpartum, 50% of the cows were cycling (TAI+NM = 18/40 and NM = 22/40). Similar percentages of cows cycling have been observed in animals gaining body weight after calving (12). Previous studies have shown that if dams are gaining body fat, as in the case of this study, the use of a hormonal treatment could be beneficial even if the calf is present (13, 14). A significant difference was observed in the number of animals with the presence of an active corpus luteum in group TAI+NM after application of the synchronization protocol, when compared against those with NM. Pessoa et al. (15) found in Bos taurus in Brazil, an increase in the number of cycling animals after application of synchronizing agents compared to females in a natural mating program. Diaz et al. (2) reported that there was no effect of the presence of the calf, or frequency of suckling, on the onset of postpartum ovarian function, suggesting, that energy loss due to the stress of lactation, in animals with a sound metabolic profile, has a minimal effect. The present study supports this concept as changes in BFT between the animals cycling vs. non-cycling by day 75 postpartum was significantly different. A similar dichotomy was observed even by the end of the experiment on day 120 postpartum.

The percentage of animals pregnant after 30 days of the breeding season was 62% for TAI+NM and for natural mating only 35%. The concept that the use of synchronization agents and timed artificial insemination, will increase the number of cows pregnant at the beginning of the breeding season in the tropics has been reported (4). However, supporting their results, at the end of the experimental trial, the number of pregnancies was similar TAI+NM 80% and NM 72%. Nonetheless, the number of pregnant animals, and their accumulative percentage through time, favored the cows that were synchronized and inseminated during the first 2 weeks. Similarly, Pessoa et al. (15) found that the number of pregnant animals varied between those who received TAI+NM (123/200 = 59.6%) and those who only were in natural mating (84/266 = 31.7%). As the number of cycling females increased, due to the synchronization protocol, this procedure eased the breeding program. The importance of having females cycling earlier in the postpartum has been reported (4).

Diaz et al. (2) comparing diverse methods of calf separation found, like other authors, that no matter what reproductive system is implemented, about 20% of the cows do not respond starting their ovarian function. As in the case of this study, 17 cows were not cycling after 120 days postpartum, which showed a mean of 0.71 ± 0.18 centimeters of backfat thickness compared to those cycling 1.04 ± 0.09 centimeters. Ayres et al. (16) found differences between backfat thickness measured through different times pre and postpartum, but no mention was given for the proportion of cows that were cycling and/or non-cycling. All these information could partially explain the difficulties in achieving a pregnancy rate in the herd on an annual basis. Rodrigues et al. (17) tested different types of resynchronizations and reported that at the end of the 105-day breeding season the pregnancy rate was very similar between treatments. However, 14–20% of the females in the treatments were not pregnant. Molina et al. (18) calculated the cost of a gestation with 52% pregnant, following diverse estrous synchronization protocols, finding that, the pregnancy cost could vary from 65 to 98 US dollars depending on the protocol. How this cost matches with only natural mating? this is a question that practitioners and farmers alike should ask themselves, before embarking in a reproductive management program.

The strong correlation between body condition score and reproductive outcomes has been addressed by several studies in the tropics (16, 19–22). However, there is a debatable issue due to inconsistencies related to the degree of technical expertise or the corporal area of the animal chosen to evaluate. This could be as thinner cows, with reasonably body reserves, erroneously, would be graded as in poor body condition. In our experience, a tangible measured such as ultrasound, should be preferential over a subjective observation based on visual scoring. In effect, Ayres et al. (23) compared the efficacy and precision of body weight, condition score, and BFT and reported a poor correlation between the former two methods and the latter. Even more, as the portability and cost of ultrasound equipment are becoming simpler and cheaper, it is not far-fetched to recommend clinicians to adopt this technique in their normal endeavors.

Escrivão et al. (24) found no differences in the weaning weights of the calves even after performing separation methods of the offspring to promote the resumption of ovarian activity. However, there is no discrimination from the month of birth and their effect on weaning weight in their study. Daily weight gain was affected by the month when the calf was born; thus, March was advantageous for daily weight gain and weight at weaning compared to calves born in April, May, or June. As far as we know, there is no data available on the direct relationship between the backfat thickness of the dam, with the performance of calves prior to weaning. Nevertheless, calf growth from birth to weaning depends on the milk supply of the dam, which could be affected by her nutritional conditions and the demand of the calf. According to Sampaio (25), the increase in the protein content of the diet will favor the intake of dry matter from low quality tropical forages. Therefore, the calves born in March, could be favored by the amount and quality of milk consumed by the dam in this month.

## Conclusions

Our findings pointed out that the most important element is the monitoring of BFT around calving for predicting the onset of ovarian activity, regardless of the breeding program utilized. However, BFT to forecast the calf growth, had an unpredictable mission to estimate their development until weaning. So, the farmer concern, should be, to have better feeding and health management during the close-up period; otherwise, despite the efforts enhancing the backfat thickness of the cows after calving, the restarting of their cyclicity could be jeopardized.

## Data Availability Statement

The raw data supporting the conclusions of this article will be made available by the authors, without undue reservation.

## Ethics Statement

The animal study was reviewed and approved by Animal Care Internal Committee of the Faculty of Veterinary Medicine and Zootechnics of the National Autonomous University of Mexico.

## Author Contributions

JM: sample collection, animal monitoring, conceptualization, data curation, and writing—original draft. CG: funding acquisition, methodology, and writing—review and editing. PO: sample collection, animal monitoring, and writing—review and editing. MC and IR: methodology, experiment execution, and writing—review and editing. JR-Z: funding acquisition, formal analysis, software, methodology, and writing—review and editing. All authors contributed to the article and approved the submitted version.

## Funding

This research received partial support from Support Program for Research and Technological Innovation Projects (PAPIIT) IN216820, National Autonomous University of Mexico.

## Conflict of Interest

The authors declare that the research was conducted in the absence of any commercial or financial relationships that could be construed as a potential conflict of interest.

## Publisher's Note

All claims expressed in this article are solely those of the authors and do not necessarily represent those of their affiliated organizations, or those of the publisher, the editors and the reviewers. Any product that may be evaluated in this article, or claim that may be made by its manufacturer, is not guaranteed or endorsed by the publisher.

## References

[1] Tinoco-Magaña JC Aguilar-Pérez CF Delgado-León R Magaña-Monforte JG Ku-Vera JC Herrera-Camacho J. Effects of energy supplementation on productivity of dual-purpose cows grazing in a silvopastoral system in the tropics. Trop Anim Health Prod. (2012) 44:1073–8. 10.1007/s11250-011-0042-8 22193937

[2] Díaz R Galina CS Rubio I Corro M Pablos JL Rodríguez A. Resumption of ovarian function, the metabolic profile and body condition in Brahman cows (Bos indicus) is not affected by the combination of calf separation and progestogen treatment. Anim Reprod Sci. (2017) 185:181–7. 10.1016/j.anireprosci.2017.08.018 28911854

[3] Rangel J Perea J De-Pablos-Heredero C Espinosa-García JA Mujica PT Feijoo M. Structural and technological characterization of tropical smallholder farms of dual-purpose cattle in Mexico. Animals. (2020) 10:1–13. 10.3390/ani10010086 31948080 PMC7023156

[4] Sa-Filho MF Penteado L Reis EL Reis TA Galvão KN Baruselli PS. Timed artificial insemination early in the breeding season improves the reproductive performance of suckled beef cows. Theriogenology. (2013) 79:625–32. 10.1016/j.theriogenology.2012.11.016 23261306

[5] Galindo J Galina CS Estrada S Romero JJ Alarcón M Maquivar M. Effect of changes in body weight, body condition and back fat during last month of pregnancy on the reproductive efficiency of Bos indicus cows in the tropics of Costa Rica. Open J Vet Med. (2013) 44:22–8. 10.4236/ojvm.2013.31005

[6] Díaz R Galina CS Rubio I Corro M Pablos JL Orihuela A. Monitoring changes in back fat thickness and its effect on the restoration of ovarian activity and fertility in Bos indicus cows. Reprod Domest Anim. (2018) 53:495–501. 10.1111/rda.13136 29356122

[7] Martínez JF Galina CS Rubio I Balam WL Corro M. Reproductive and cost assessment of a seasonal breeding program with Bos indicus in tropical Mexico. Rev MVZ Cordoba. (2020) 26:1–10. 10.21897/rmvz.2130

[8] Schröder UJ Staufenbiel R. Invited review: Methods to determine body fat reserves in the dairy cow with special regard to ultrasonographic measurement of backfat thickness. J Dairy Sci. (2006) 89:1–14. 10.3168/jds.S0022-0302(06)72064-1 16357263

[9] Bisinotto R. S. Chebel R. C. Santos J. E. P. (2010). Follicular wave of the ovulatory follicle and not cyclic status influences fertility of dairy cows. J. Dairy Sci. 93 3578–3587. 10.3168/jds.2010-3047 10.3168/jds.2010-3047 20655426

[10] Diskin MG Kenny DA. Managing the reproductive performance of beef cows. Theriogenology. (2016) 86:379–87. 10.1016/j.theriogenology.2016.04.052 27180327

[11] Sinclair KD Molle G Revilla R Roche JF Quintans G Marongiu T. Ovulation of the first dominant follicle arising after day 21 post-partum in suckling beef cows. Anim Sci. (2002) 75:115–26. 10.1017/S135772980005289930886898

[12] Mulliniks JT Cox SH Kemp ME Endecott RL Waterman RC VanLeeuwen DM. Relationship between body condition score at calving and reproductive performance in young postpartum cows grazing native range. J Anim Sci. (2012) 90:2811–7. 10.2527/jas.2011-4189 22665663

[13] Pérez-Torres L Orihuela A Corro M Rubio I Cohen A Galina CS. Maternal protective behavior of zebu type cattle (Bos indicus) and its association with temperament. J Anim Sci. (2014) 92:4694–700. 10.2527/jas.2013-7394 25149346

[14] Baruselli PS Ferreira RM Sa-Filho MF Bó GA. Using artificial insemination v. natural service in beef herds. Animal. (2018) 12:45–52. 10.1017/S175173111800054X 29554986

[15] Pessoa GA Martini AP Sa-Filho MF Rubin MIB. Resynchronization improves reproductive efficiency of suckled Bos taurus beef cows subjected to spring-summer or autumn-winter breeding season in South Brazil. Theriogenology. (2018) 122:14–22. 10.1016/j.theriogenology.2018.08.021 30199740

[16] Ayres H Ferreira RM Torres-Júnior JRS Demétrio CGB Sa-Filho MF Gimenes L. Inferences of body energy reserves on conception rate of suckled Zebu beef cows subjected to timed artificial insemination followed by natural mating. Theriogenology. (2014) 82:529–36. 10.1016/j.theriogenology.2014.04.026 24969365

[17] Rodrigues WB do Prado Jara J Borges JC de Oliveira LOF de Abreu UPG Anache NA. Efficiency of mating, artificial insemination or resynchronisation at different times after first timed artificial insemination in postpartum Nellore cows to produce crossbred calves. Anim. Prod. Sci. (2019) 59:225–31. 10.1071/A.N.1746628948418

[18] Molina JJ Molina I Jiménez A Galina CS Romero JJ. Pharmacological control of estrus in tropical cattle, an economical assessment of different synchronization protocols. Open J Vet Med. (2012) 2:151–7. 10.4236/ojvm.2012.23024

[19] Montiel F Ahuja C. Body condition and suckling as factors influencing the duration of postpartum anestrus in cattle: a review. Anim Reprod Sci. (2005) 85:1–26. 10.1016/j.anireprosci.2003.11.001 15556305

[20] Fernandes AFA Neves HHR Carvalheiro R Oliveira JA Queiroz SA. Body condition score of Nellore beef cows: a heritable measure to improve the selection of reproductive and maternal traits. Animal. (2015) 9:1278–84. 10.1017/S1751731115000154 25703049

[21] Mondragón V Galina CS Rubio I Corro M Salmerón F. Effect of restricted suckling on the onset of follicular dynamics and body condition score in Brahman cattle raised under tropical conditions. Anim Reprod Sci. (2016) 167:89–95. 10.1016/j.anireprosci.2016.02.011 26936657

[22] Pfeifer LFM Rodrigues WB Nogueira E. Relationship between body condition score index and fertility in beef cows subjected to timed artificial insemination. Livest Sci. (2021) 248:1–5. 10.1016/j.livsci.2021.104482

[23] Ayres H Ferreira RM Torres-Júnior JRS Demétrio CGB de Lima CG Baruselli PS. Validation of body condition score as a predictor of subcutaneous fat in Nelore (Bos indicus) cows. Livest Sci. (2009) 123:175–9. 10.1016/j.livsci.2008.11.004

[24] Escrivão RJA Webb EC Garcês APDJT Grimbeek RJ. Effects of 48-hour calf withdrawal on conception rates of Bos indicus cows and calf weaning weights in extensive production systems. Trop Anim Health Prod. (2012) 44:1779–82. 10.1007/s11250-012-0137-x 22528530

[25] Sampaio CB Detmann E Paulino MF Valadares Filho SC de Souza MA Lazzarini I. Intake and digestibility in cattle fed low-quality tropical forage and supplemented with nitrogenous compounds. Trop Anim Health Prod. (2010) 42:1471–9. 10.1007/s11250-010-9581-7 20414721

